# Modulation of gonadotrophin induced steroidogenic enzymes in granulosa cells by d-chiroinositol

**DOI:** 10.1186/s12958-016-0189-2

**Published:** 2016-08-31

**Authors:** Sandro Sacchi, Federica Marinaro, Debora Tondelli, Jessica Lui, Susanna Xella, Tiziana Marsella, Daniela Tagliasacchi, Cindy Argento, Alessandra Tirelli, Simone Giulini, Antonio La Marca

**Affiliations:** 1 Mother-Infant Department, University of Modena and Reggio Emilia, Via del pozzo 41, 41100 Modena, Italy; 2 University of Modena and Reggio Emilia and Clinica Eugin Modena, Modena, Italy

**Keywords:** d-chiroinositol, Steroiodogenesis, Insulin resistance

## Abstract

**Background:**

d-chiroinositol (DCI) is a inositolphosphoglycan (IPG) involved in several cellular functions that control the glucose metabolism. DCI functions as second messenger in the insulin signaling pathway and it is considered an insulin sensitizer since deficiency in tissue availability of DCI were shown to cause insulin resistance (IR). Polycystic ovary syndrome (PCOS) is a pathological condition that is often accompanied with insulin resistance. DCI can positively affects several aspect of PCOS etiology decreasing the total and free testosterone, lowering blood pressure, improving the glucose metabolism and increasing the ovulation frequency. The purpose of this study was to evaluate the effects of DCI and insulin combined with gonadotrophins namely follicle-stimulating hormone (FSH) and luteinizing hormone (LH) on key steroidogenic enzymes genes regulation, *cytochrome P450 family 19 subfamily A member 1* (*CYP19A1*) and *cytochrome P450 side-chain cleavage* (*P450scc*) in primary cultures of human granulosa cells (hGCs). We also investigated whether DCI, being an insulin-sensitizer would be able to counteract the expected stimulator activity of insulin on human granulosa cells (hGCs).

**Methods:**

The study was conducted on primary cultures of hGCs. Gene expression was evaluated by RT-qPCR method. Statistical analysis was performed applying student *t*-test, as appropriate (*P* < 0.05) set for statistical significance.

**Results:**

DCI is able to reduce the gene expression of *CYP19A1*, *P450scc* and *insulin-like growth factor 1 receptor* (*IGF-1R*) in dose–response manner. The presence of DCI impaired the increased expression of steroidogenic enzyme genes generated by the insulin treatment in gonadotrophin-stimulated hGCs.

**Conclusions:**

Insulin acts as co-gonadotrophin increasing the expression of steroidogenic enzymes genes in gonadotrophin-stimulated granulosa cells. DCI is an insulin-sensitizer that counteracts this action by reducing the expression of the genes *CYP19A1*, *P450scc* and *IGF-1R.* The ability of DCI to modulate in vitro ovarian activity of insulin could in part explain its beneficial effect when used as treatment for conditions associated to insulin resistance.

## Background

The cis-1, 2, 4-trans-3, 5, 6-Cyclohexanehexol, also known as d-chiroinositol (DCI), is a six-carbon polyalcohol which belongs to the family collectively referred to as inositol which is a part of the B vitamin family. At cellular level inositols are incorporated into cell membranes as phosphatidyl-myo-inositol, the precursor of inositol triphosphate which acts as second messenger and regulates the activities of different hormones such as follicle-stimulating hormone (FSH) and insulin [1]. Epimerase enzymes convert inositols to up to nine stereoisomers including myo-inositol (MI) and DCI while the majority of the other stereoisomers generated fail to show an evident biological activity [2]. MI and DCI are both endogenous biosynthesized and introduced from dietary sources such as buckwheat [3], *Cucurbita ficifolia* [4] and soy lecithin [5].

DCI is considered as an insulin sensitizer since inositolphosphoglycan (IPG) mediators are involved in several cellular functions that control the glucose metabolism [6, 7].

Moreover, impaired metabolism of IPG mediators as well as a deficiency in tissue availability of inositol were shown to cause insulin resistance [8, 9].

Since DCI is synthetized by an epimerase that converts in vivo MI to DCI, several studies observed that a decreased DCI in urine as well as tissues of human subjects and animals with type 2 diabetes was accompanied by an increase in MI content [10, 11].

Additional investigations demonstrated that the altered inositol excretion patterns in human and monkey urine were specifically related to the underlying insulin resistance (IR), rather than to the diabetes type. To explain the altered pattern of urine inositol excretion observed under IR, a defect in the epimerization process was hypothesized.

When IR occurs, the conversion rate is affected, resulting in a decreased level of DCI in cells.

In 2003 the European Society for Human Reproduction and Embryology (ESHRE) and the American Society for Reproductive Medicine (ASRM) established that patients can be affected by polycystic ovary syndrome (PCOS) when at least two conditions as anovulation or hyperandrogenism or increased ovarian volume are verified at the same time. However, PCOS patients are often affected by IR and it was hypothesized that a DCI deficiency, which functions as second messanger in the insulin signaling pathway [12], can be related to IR [13].

It has been demonstrated that DCI can positively affects several aspect of PCOS etiology [8, 14]. In these studies DCI was able to decrease the total and free testosterone, to lower blood pressure acting as insulin sensitizer by improving the glucose metabolism, and finally to increase the ovulation frequency [8, 14, 15].

Insulin has been reported to be able to interact with steroidogenic enzymes in granulosa and luteinic cells of the ovaries [16–20]. Specifically insulin seems to potentiate the FSH and luteinizing hormone (LH) induction of *cytochrome P450 family 19 subfamily A member 1* (*CYP19A1*) aromatase and this effect has been shown to be so evident that insulin is considered by many as a co-gonadotrophin [17–19, 21]. The co-treatment, in vitro, with insulin-sensitizers, such as metformin or thiazelinediones, counteracts the potent action of insulin on human granulosa cells (hGCs) enzymes hence showing the relevance of insulin activity for ovarian cells physiology [16–21].

The objective of this study was to expand on this argument, hence investigating the possible effect of insulin on the ovarian response to both the two gonadotrophins FSH and LH. We specifically investigated the modifications of the gene expression of two key genes in steroidogenesis, the aromatase *CYP19A1* and *cytochrome P450 side-chain cleavage* (*P450scc*). We also evaluated whether DCI, being an insulin-sensitizer would be able to counteract the expected stimulator activity of insulin on hGCs.

## Methods

### Selection criteria

Patients selected (*n* = 8) for this study were heathy, had regular menstrual cycles and had a mean age of 34 (±8). Patients underwent in vitro fertilization (IVF) because of male infertility. Clinical exclusion criteria adopted were: previous ovarian surgery, positivity for Chlamydia antibody testing (CAT), presence of ovarian cysts, history of pelvic inflammatory disease (PID), any known metabolic or endocrinological disease.

Patients underwent IVF cycles according the gonadotrophin-releasing hormone (GnRH) antagonist protocol. Patients received recombinant human FSH (rhFSH, Gonal F®, Merck Serono, Italy or Puregon®, MSD Organon, Italy) at a dose of at least 150 international units (IU) a day subcutaneously from day 2 or 3 of a spontaneous menstrual cycle. The GnRH antagonist, Ganirelix (Orgalutran, Schering-Plough) or Cetrorelix (Cetrotide, Merck Serono, Italy), was next administered daily by subcutaneous injection (0.25 mg/d) from the day of the stimulation cycle when the first follicle reached 14 mm in size to the day of human chorionic gonadotrophin (hCG) administration. When follicles reached ≥18 mm, 10000 IU of hCG were administrated intramuscularly and 34–36 h later follicles were aspirated under patient sedation. hGCs were isolated from ovarian follicles of women undergoing oocyte retrieval for IVF protocol. Study approval was obtained from the local ethics committee and informed, written consent was obtained from each patient.

### Granulosa-lutein cell isolation and primary cell culture

hGCs were purified by centrifugation through a discontinuos Percoll (Amersham, Sweden) gradient as indicated in [22], and cultured individually in 24 wells plate (50 x 10^3^ cells/well) in McCoy 5A medium (Carlo Erba, Italy) supplemented with 5 % foetal bovine serum (FBS) South America (EU Approved, Carlo Erba, Italy), 2mM L-Glutamine, 1 % Penicillin/Streptomicin and 1 % Amphotericin B (Sigma Aldrich, St. Louis, MO, USA). Primary hGCs culture were mantained at 37 °C under a controlled atmosphere of 5 % CO_2_ for 6 days to avoid side effect due to IVF hormones treatment, and subjected to medium changes with fresh culture medium daily.

### Treatments

hGCs primary cultures were initially incubated in starvation medium (McCoy 5A medium supplemented with 0.1 % FBS South America without antibiotics) for 12 h to synchronize cells before the treatments. hGCs were then incubated for 24 h with 0,1 IU/ml of long-acting insulin (Levemir®, Novo Nordisk, Denmark). hGCs insulin-treated where additionally incubated with 20 nM or 10 nM DCI (LJ Pharma, Italy) alone or in combination with either 5 μM rhFSH, (Gonal-F®, Merck Serono, Italy) or 5 μM of recombinant human LH (rhLH, Luveris®, Merck Serono, Italy) for further 24 h. DCI and gonadotrophins were dissolved into starvation medium. Experiments were repeated in triplicate. Negative controls using corresponding amount of vehicle control for insulin treatment and for each of gonadotrophin used were performed. Comparison between negative controls performed and untreated cells showed no differences in terms of cells vitality, toxic effects or impaired gene expression caused directly by the vehicle in which substances were dissolved.

### Trypan blue exclusion test of cell viability

To assess the viability of gonadotrophin treated hGCs primary cultures, the Trypan Blue (Sigma Aldrich, St. Louis, MO, USA) exclusion test was performed. hGCs corresponding to each treatment and controls were submerged in 0.4 % Trypan Blue in 1X Dulbecco’s phosphate buffered saline (DPBS, Sigma Aldrich, St. Louis, MO, USA) solution directly in the wells then immediately counted (*n* = 200) under inverted microscope PrimoVert (Carl Zeiss Microscopy GmbH, Germany). Percentage of viable cells were calculated and expressed as ratio between blue-stained dead cells and not colored viable cells.

### Evaluation of gene expression by RT-qPCR

Collected hGCs after each treatment were immediately processed for total RNA extraction using commercial product Tri-Reagent® (Sigma Aldrich, St Louis, MO, USA) following the manufacturer protocol. After an optional DNase I (Promega, Madison, WI, USA) digestion of 30 min at 37 °C the extracted RNA was evaluated and quantified by spectrophotometry using Nanodrop ND-1000 (Thermo Fisher Scientific, Waltham, MA, USA) and 2 μg of RNA of each sample was reverse transcribed to cDNA using iScript™ cDNA Synthesis Kit (Bio-Rad, Hercules, CA, USA) according to datasheet. 2 μL of cDNA of each sample was tested in triplicate in RT-qPCR using SsoAdvanced Universal SYBR® Green Supermix (Bio-Rad, Hercules, CA, USA) following the conditions suggested by the manufacturer’s protocol. Primers used (listed in Table 1) in RT-qPCR reaction were designed, where possible, across the intron to avoid gDNA contaminants amplification. All primers used has similar melting temperature so the general thermal profile of the reaction is for all the genes tested 30 s at 95 °C to initially activate the enzyme followed by 40 cycles of 95 °C for 5 s and 60 °C for 20 s for each cycle. Results were normalized by using the reference gene *Ribosomal protein S7* (*RpS7*) gene. The specificity of each assay was validated by dissociation curve analysis and amplicons were separated by gel electrophoresis and imaged. Assay performance was validated by evaluating amplification efficiencies by means of calibration curves, and ensuring that the plot of log input amount versus DCq (also known as DCT) has a slope. Each reaction was repeated in triplicate. Negative control reactions omitted templates.

**Table 1:** Primers list used in RT-qPCR

Gene	Protein name	Sequence 5′- 3′	Amplicon lenght (bp)	NCBI Ref. sequence
*RpS7*	Ribosomal protein S7	F: AATCTTTGTTCCCGTTCCTCA	135	NM_001011.3
		R: CGAGTTGGCTTAGGCAGAA		
*β3 integrin*	β3 integrin	F: GACAAGGGCTCTGGAGACAG	233	NM_000212.2
		R: ACTGGTGAGCTTTCGCATCT		
*IGF1R*	Insulin-like growth factor 1 receptor	F: CGTGGGAGGGTTGGTGATTA	161	NM_000875.3
		R: TGGCCACTCTGGTTTCAGGT		
*CYP19A1*	Aromatase	F: CCCTTCTGCGTCGTGTCAT	86	NM_000103.3
		R: GATTTTAACCACGATAGCACTTTCG		
*P450scc (CYP11A1)*	Cholesterol side-chain cleavage enzyme	F: ACCAAGAACTTTTTGCCCCT	127	NM_000781.2
		R: ATGTCCCCCGAGTAATTTCC		

### Statistical analysis

Statistical analysis was performed applying student *t*-test, as appropriate (*P* < 0.05) set for statistical significance. The relative expression of each gene has been evaluated using the 2^-ΔΔCt^ method [23] and was calculated as the relative ratio in comparison to the first control sample, set arbitrarily to 1.

## Results

**Fig. 1:**
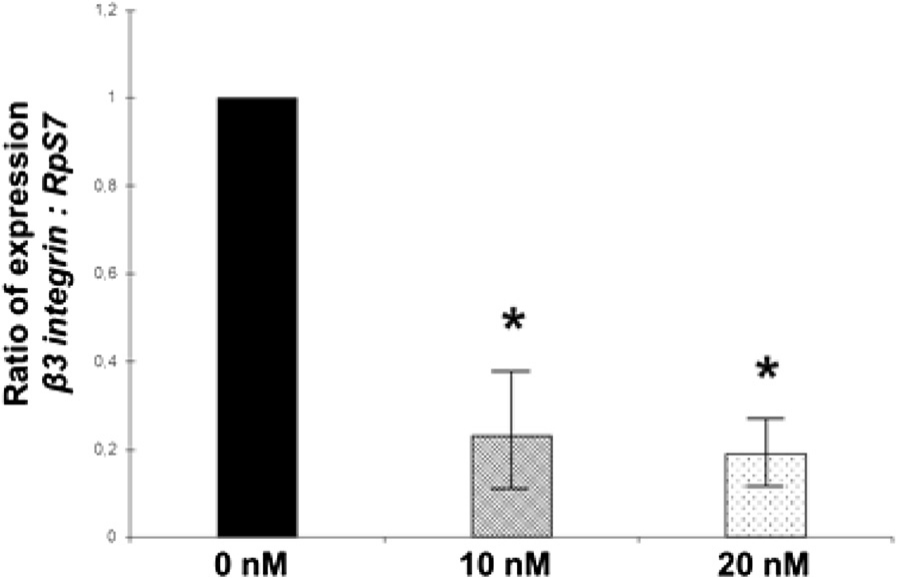
Effect of 24 h incubation with increasing dosage of DCI, range 0 nM - 20 nM, on *β3 integrin* gene expression normalized by the reference *RpS7* gene in primary culture of hCGs in vitro by RT-qPCR. Significant differences versus the respective controls were marked by * *p* < 0.05, Student’s *t*-test

To investigate a possible role of DCI as modulator of gene expression, hGCs primary culture were treated for 24 h with increasing concentrations of DCI dissolved into starvation medium. In order to assess the cell viability at the end of the DCI treatment the Trypan Blue exclusion test was performed while the *β3 integrin* gene activation, expressed as ratio normalized by *RpS7* reference gene on RT-qPCR assay, was studied as positive control (Fig. 1).

At all concentrations of DCI tested the calculated precentage of viable hGCs was higher than 95 % without any remarkable difference among treatments and in comparison to the untreated hGCs control (data not shown). As Fig. 1 shows, DCI is able to directly decrease the *β3 integrin* gene expression of hGCs in a dose–response manner. DCI-treated primary cultures of hGCs that failed to show decrease of *β3 integrin* gene expression were excluded from further analysis.

### d-chiroinositol affects the steroidogenic enzymes gene expression

**Fig. 2:**
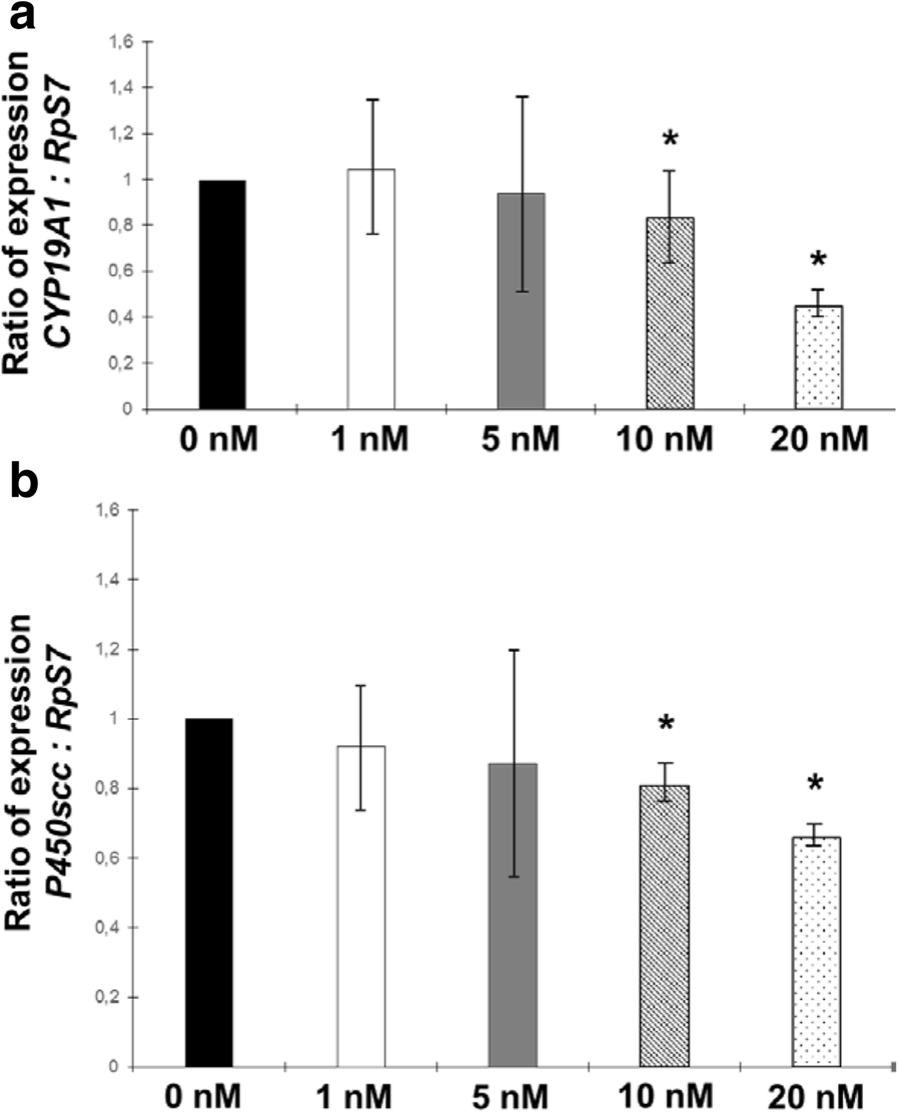
Evaluation of dose–response effect of 24 h incubation with DCI on **a** aromatase *CYP19A1* and **b** *P450scc* genes expression in primary culture of hGCs by RT-qPCR. Significant differences versus the respective controls were marked by * *p* < 0.05, Student’s *t*-test

The effect of 24 h incubation with increasing dosages of DCI on steroidogenic enzymes gene activation in primary cultures of hGCs was studied by RT-qPCR and expressed as ratio normalyzed by *RpS7* reference gene. Figure 2 shows the dose–response curve generated by different concentrations of DCI on *CYP19A1* (Fig. 2a) and *P450scc* (Fig. 2b) gene expression.

DCI has tendency to decrease the expression of both genes analyzed at the lowest concentrations tested and this effect is particularly significative with higher DCI dosages which were choosen and employed for following experiments.

### The insulin up-regulation of the steroidogenic enzyme genes response to gonadotrophins is balanced by d-chiroinositol treatment

**Fig. 3:**
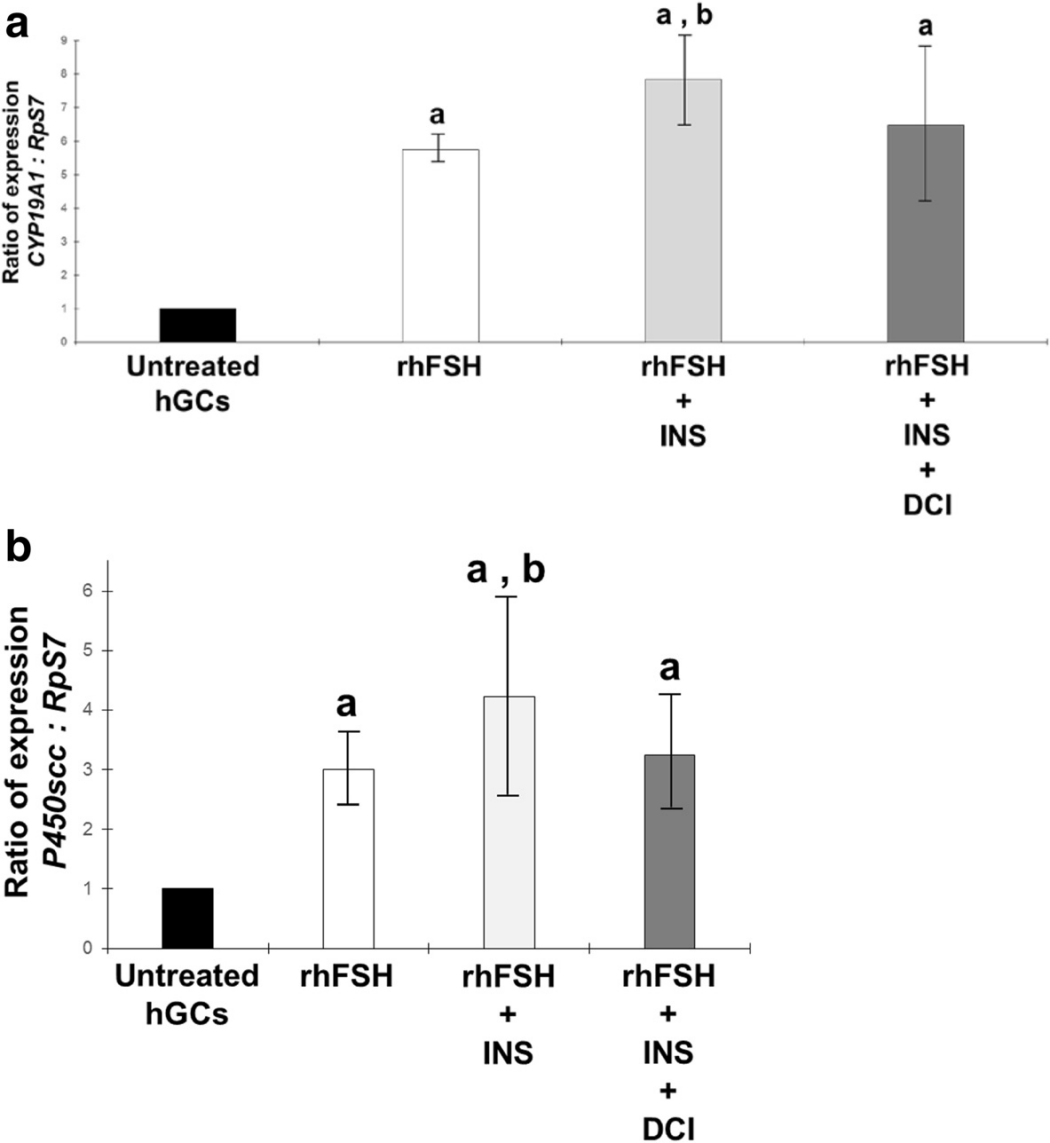
Effect of 24 h incubation with 5 ng/ml rhFSH on **a** *CYP19A1* and **b** *P450scc* gene expression alone, and in combination with 0,1 U insulin or 20 nM DCI or both in primary culture of hGCs cells in vitro. Different letters indicate different significances at *p* < 0.05, Student’s *t* test

**Fig. 4:**
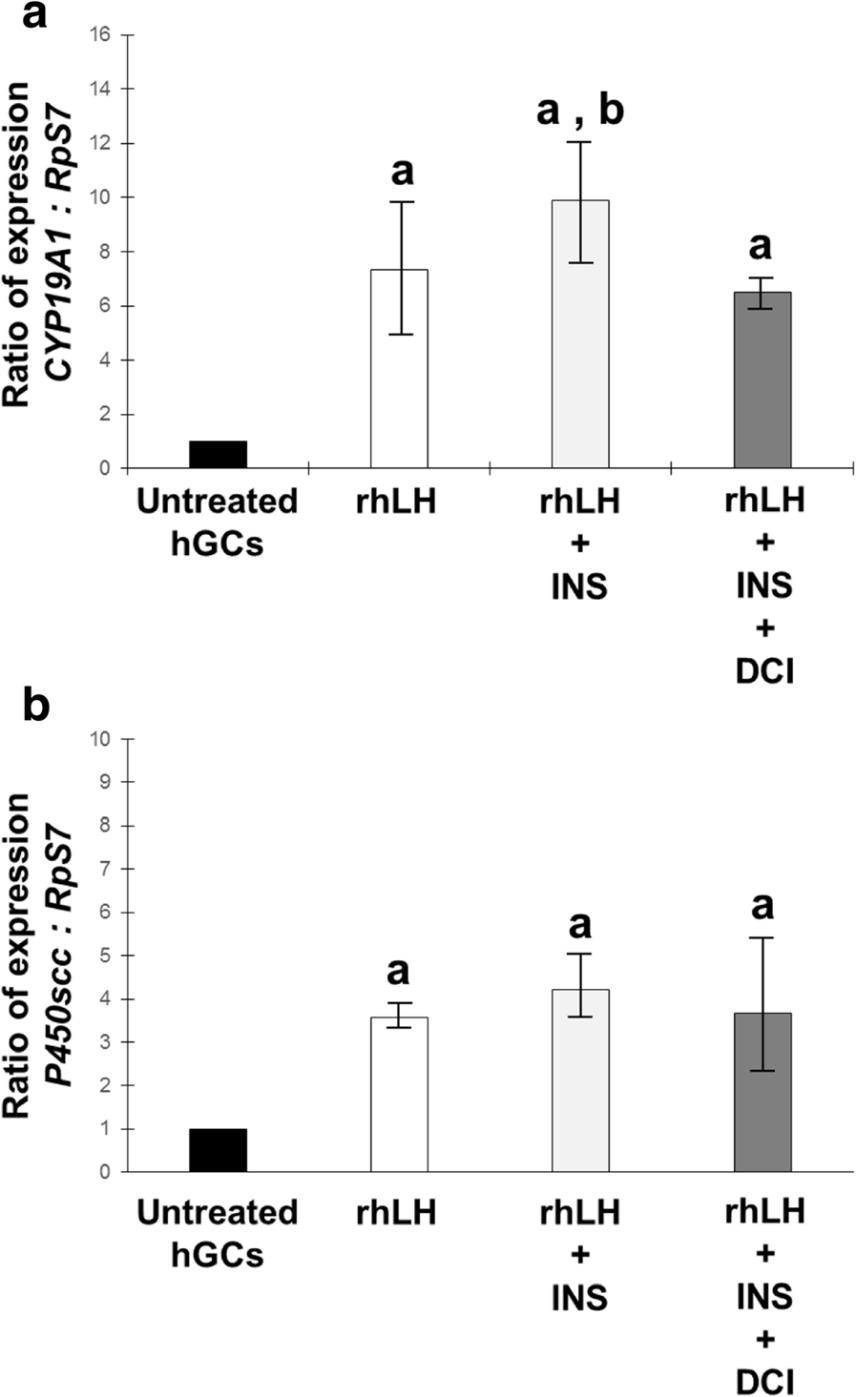
Effect of 24 h incubation with 5 ng/ml rhLH on **a** *CYP19A1* and **b** *P450scc* gene expression alone, and in combination with 0,1 U insulin or 20 nM DCI or both in primary culture of hGCs in vitro. Different letters indicate different significances at *p* <0.05, Student’s test

**Fig. 5:**
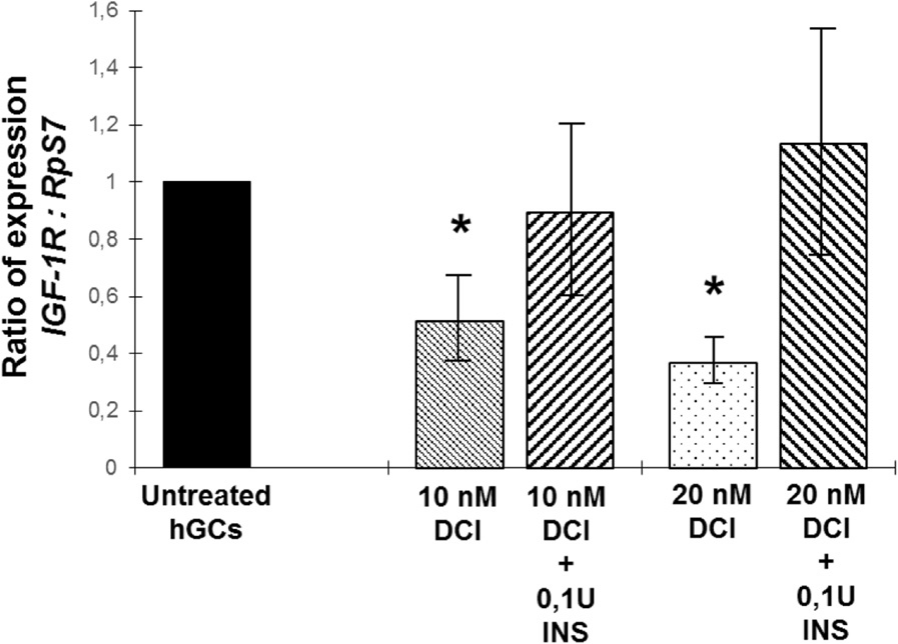
Dose–response effect of 24 h of incubation with 10 nM or 20 nM DCI alone or in combination with 0,1 U insulin on *IGF1R* gene expression in primary culture of hGCs in vitro by RT-qPCR. Significant differences versus untreated controls were marked by * *p* < 0.05, Student’s *t*-test

Primary cultures of hGCs were treated with gonadotrophins alone and in combination with 0,1 IU/ml insulin or 20 nM DCI or both. Steroidogenic enzymes gene expression was calculated by RT-qPCR reactions and expressed as ratio normalyzed by *RpS7* refrence gene. The addition of 5 ng/ml rhFSH (Fig. 3) or 5 ng/ml rhLH (Fig. 4) was associated to a significant activation of gene expression of both *CYP19A1* and *P450scc*. Expectantly, the co-incubation of gonadotrophins with 0,1 IU/ml of insulin resulted significantly more effective in inducing the two genes when compared to rhFSH alone (Fig. 3a, b) and rhLH alone (Fig. 4a). When added in the cultural system, the samples incubated with 20 nM DCI show no significative differences if compared with the effect generated by gonadotrophin alone treatment (Figs. 3 and 4). Meanwhile, the positive contribution of insulin to the steroidogenic genes induction generated by gonadotrophins is impaired by DCI co-incubation.

Finally both concentrations of DCI tested (10 nM or 20 nM) revealed that DCI is able to directly reduce *insulin-like growth factor 1 receptor* (*IGF-1R*) gene expression while the co-incubation of DCI and 0,1 U of insulin in the culture system is not producing significatively differences between treated and untreated hGCs controls (Fig. 5).

## Discussion

In the present study we demonstrated that insulin and DCI are able to affect directly the gene expression of two pivotal steroidogenic enzymes gene namely *CYP19A1* and *P450scc*. Moreover, DCI may modulate the stimulatory effects on steroidogenic genes mediated by both gonadotrophins and insulin.

The efficiency of the cultural system in which DCI treatments affected the genes regulation in hGCs primary cultures was evaluated trough the analysis of the expression of *β3 integrin* gene. Integrins are membrane receptors involved in cells-extracellular matrices adhesion and in the signal transduction that are differentially and stage-specifically expressed on hGCs [24]. It was shown that integrins are able to regulate different hGCs functions [25] as well as the corpus luteum formation [26, 27]. For these reasons, it was hypothesized a possible role of integrins during folliculogenesis [28–30]. Moreover, in a different study conducted to evaluate a possible anti-metastasis effects of D-pinitol, a 3-methoxy DCI analogue, in human prostate cancer cells, it was demonstrated that noncytotoxic concentrations of D-pinitol reduced mRNA and cell surface expression of the β3 integrin fraction on prostate cancer cells [31]. Based on the percentage of viable cells after the Trypan Blue exclusion assay, increasing concentrations of DCI were able to reduce the *β3 integrin* gene expression in all the hGCs primary cultures employed in this study.

In addition, our analyses show that DCI acts directly on steroidogenic enzymes gene regulation of hGCs reducing mRNA expression of both *CYP19A1* and *P450scc* genes in dose–response manner. P450scc enzymes catalyze many reactions involved in synthesis of cholesterol and steroids and togheter with the aromatase CYP19A1 are required to regulate estradiol and progesterone production within the ovary [32].

However, because the mature granulosa-lutein cells used for this in vitro study may not reflect the in vivo condition, the experiments were also conducted in presence of gonadotrophins and insulin. Both the gonadotrophins (alone or in combination) induce expression of *CYP19A1* and *P450scc* genes [33, 34] although gonadotrophin-related profile of gene expression exists [35, 36]. It is also widely demonstrated that insulin plays a role in the ovarian functions by enhancing the gonadotrophin effects as showed in theca cells [37, 38] and in granulosa cells [39, 40]. In our experimental model, incubation with rhFSH or rhLH were able to induce *CYP19A1* and *P450scc* gene expression according with previously published data [36]. Moreover, our data confirmed that insulin could influence steroidogenesis by increasing the stimulatory effects of LH and FSH, as showed in our study, by increasing the expression of steroidogenic enzyme genes. Interestingly the positive effect of insulin on gonadotrophin-stimulated hGCs was counter-acted by the presence of DCI. However, the hGCs cultured in presence of DCI were still responsiveness to gonadotrophins as the *CYP19A1* and *P450scc* were significatively induced if compared to the untreated hGCs control.

Since it was also reported that DCI plays a role as second messengers in the classical phosphorylation signaling pathways activated by insulin triggering the activation of Mg^2+^-dependent protein phosphatases (MPPs) family which control the glucose metabolism [5], we investigate the effects of co-treatment with DCI and insulin in hGCs.

Notoriously, insulin exerts its function not only by binding insulin receptors present on the surface of target cells but also functioning as ligand for IGF-1R [41]. The typical ligand for IGF-1R is the hormone insulin-like growth factor (IGF) that is considered to have co-gonadotrophin effects.

In the last years one of the most studied of the in vivo pathology related to infertility was the PCOS. The PCOS is characterized by elevated levels of insulin and IGF-1R [42] and it has been hypothesized that this could influence the pathogenesis of the syndrome and the alterations of sexual steroids commonly frequent in patients affected by PCOS. IGF-1R is present on the cellular membrane of hGCs [43] and it has been demonstrated that different insulin sensitizer drugs such metformin are able to modulate gene expression [20, 44].

In the present study, we showed not only that DCI is able to balance the additional stimulatory effect on steroidogenic enzyme gene of gonadotrophins stimulated hGCs, but also we firstly demonstrated that incubation of hGCs with increasing dosage of DCI resulted in a decreased *IGF-1R* expression in a dose-dependency manner.

## Conclusions

In conclusion, in the present study we could confirm the effect of insulin as co-gonadotrophin on granulosa cells from the ovaries and we could demonstrate that DCI, an insulin-sensitizers counteract this action. This was evident when the expression of several genes was investigated, such as aromatase *CYP19A1*, *P450scc* and *IGF-1 receptor*. The ability of DCI to modulate in vitro ovarian activity of insulin could at least in part explain its beneficial effect when used as treatment for conditions associated to insulin-resistance.

## Abbreviations

ASRM: American Society for Reproductive Medicine
CAT: *Chlamydia* antibody testing
CYP19A1: Cytochrome P450 family 19 subfamily a member 1
P450scc: Cytochrome P450 side-chain cleavage
DCI: d-chiroinositol
DPBS: Dulbecco’s phosphate buffered saline
ESHRE: European Society for Human Reproduction and Embryology
FBS: Foetal bovine serum
FSH: Follicle-stimulating hormone
GnRH: Gonadotrophin-releasing hormone
hCG: Human chorionic gonadotrophin
hGCs: Human granulosa cells
IVF: In vitro fertilization
IPG: Inositolphosphoglycan
IR: Insulin resistance
IGF: Insulin-like growth factor
IGF-1R: Insulin-like growth factor 1 receptor
IU: International units
LH: Luteinizing hormone
MI: Myo-inositol
MPP: Mg^2+^-dependent protein phosphatase
PID: Pelvic inflammatory disease
PCOS: Polycystic ovary syndrome
rhFSH: Recombinant human FSH
rhLH: Recombinant human LH
RpS7: Ribosomal protein S7
